# Supporting open science at *PLOS Biology*

**DOI:** 10.1371/journal.pbio.3002516

**Published:** 2024-01-29

**Authors:** Lauren Cadwallader, Nonia Pariente

**Affiliations:** 1 Public Library of Science, San Francisco, California, United States of America; 2 Public Library of Science, Cambridge, United Kingdom

## Abstract

Open science is key to PLOS Biology’s mission, both in its daily operations and in the role we aspire to have in the scholarly ecosystem. In this Editorial, we reflect on open science at the journal and discuss how and why we shall continue to hold it central to everything we do.

## Teaser

Open science is key to *PLOS Biology’s* mission, both in its daily operations and in the role we aspire to have in the scholarly ecosystem. Here, we reflect on open science at the journal and discuss how and why we shall continue to hold it central to everything we do.

Open science has always been a key tenet of PLOS journals, initially with open access to publications (free access to every published article for everyone) being the focus. However, the broader concept of open science, understood as making all components of the research process (from data to code or the peer-review process) openly available, has a key role in accelerating rigorous scientific discovery, and should thus be recognized, supported, and enabled by the many stakeholders—including researchers, funders, institutions, and publishers—involved in research. Over the years, *PLOS Biology* has supported the move towards open science in many ways, for example, by introducing a data availability policy in 2014 requesting that all data necessary to replicate a study’s findings be published; integrating with bioRxiv to facilitate preprint posting by authors on submission; changing authorship guidelines to allow recognition of individuals who made crucial contributions, such as data analysis, data curation, methodology, or software development, even if they did not contribute to the writing of the article; and introducing the Accessible Data feature, an icon that marks studies with data in popular repositories and provides a direct link to the associated dataset, so it is more visible to readers.

An important part of driving open science practices is understanding the current adoption levels among different subdisciplines within our journal scope, as well as the barriers that individuals still might face, despite the emergence of technological solutions and the increasing number of funders and institutions that encourage or require these practices. Only by understanding the state of play are we able to continue to innovate in ways that work for the community.

The open science principles that are key to PLOS’ publishing mission extend to other work PLOS is doing to drive a broader change in scholarly communications, beyond its journals. One of the resources the organization has developed is the Open Science Indicators (OSI) dataset [1], which analyzes a set of open science practices in over 103,000 PLOS articles and 21,000 comparable articles from other publishers. This dataset provides insights into the sharing of data, code, and preprints since 2018, allowing the user to, for example, assess the impact of initiatives designed to increase the adoption of open science, or how different communities practice open science. We have used the OSI dataset, as well as other internal data, to understand how open science practice by *PLOS Biology* authors has evolved, and found the following:

- 100% of articles openly share data.
- The proportion of articles sharing data in a repository, considered best practice according to the FAIR principles [2], has increased from 38% in 2018 to 58% in 2023 (up to September).
- 65% of articles published in 2023 were associated with a preprint, up from 28% in 2018.
- Code sharing rates have been steadily increasing to 40% of all published papers in 2023.
- 29% of articles published since 2019 linked to one or more detailed methods protocols; 87% of these protocols were used to generate the reported results, while 13% were used to evidence background claims.
- 63% of articles published in 2023 shared their peer review history, including editor decision letters, reviewer reports, and author responses; this proportion has remained fairly stable since 2020.

The rates of adoption of open science practices seen at the journal are above those seen in the OSI comparator group, which is composed of articles from the same disciplines that PLOS publishes from the same time period. This data, for example, shows the preprint posting rate in the comparator group to be 30% and code sharing 9%; both significantly lower than the *PLOS Biology* rate. Over the past 6 years, we have made measurable progress towards fostering a community that embraces open science, in keeping with our mission of driving positive change in research publishing, and we will continue to evolve policies that drive increased adoption of these practices. Doing this type of analysis underscored the importance of strong policies and the role of editors in driving changes in practice. For example, during the summer of 2022, after realizing that an increasing number of authors deposited data in GitHub, which does not provide archiving (meaning that the information could be silently changed after publication) or a DOI, our team started asking authors to move the data (and code, if applicable) to a policy-compliant repository before an article was accepted, suggesting that authors might want to use the Zenodo integration with GitHub for its ease of use. Since then, we have seen an increase in the use of Zenodo for data sharing, concomitant with a slight decrease in the use of Github, suggesting authors are willing to adopt better open science practices when advised how to do so. For code, authors may choose to share links to both versions, as sharing in Github is a community norm that facilitates the improvement of code and sharing updated versions [3].

Although *PLOS Biology* can be proud of its contribution to open science, as we’ve discussed previously [4] we can and will do more, both in terms of signaling reliability and trustworthiness in our published research, and in providing a platform for the publication of research aiming to change current practices and mindsets. For example, we regularly cover new approaches to research assessment [5], research reporting [6], measurement of open science practices [7] (which informed the development of OSIs by PLOS), or biases within the scholarly system, such as the costs of being a non-native English speaker in science [8], editorial bias in biomedical journals [9], hurdles faced by mothers [10] or early career researchers [11], as well as discussions about the possible futures of scholarly publishing.

The beginning of the year is a time to reflect and make new plans. After the analysis of our open science performance in the OSI dataset, we have set ourselves the goal of increasing the sharing of code and publication of peer-review files in 2024. We are currently discussing how to best approach these goals. Stay tuned for our reports on progress and thank you for being part of our community of open science champions.
